# A Systematic Review of Adolescent Flourishing

**DOI:** 10.5964/ejop.6831

**Published:** 2023-02-28

**Authors:** Nicole C. Waigel, Viviana N. Lemos

**Affiliations:** 1 Centro Interdisciplinario de Investigaciones en Ciencias de la Salud y del Comportamiento (CIICSAC), Universidad Adventista del Plata. Libertador San Martín, Entre Ríos, Argentina; 2 Consejo Nacional de Investigaciones Científicas y Técnicas (CONICET), Buenos Aires, Argentina; University of Wroclaw, Wroclaw, Poland

**Keywords:** systematic review, well-being, adolescence, flourishing

## Abstract

Traditionally, the study of well-being has been approached from the hedonic and eudaimonic perspectives. However, the last findings suggest that both aspects are complementary, giving place to an integrated conceptualization of well-being called flourishing. In spite of the constant increase of research around this construct, there is still little information regarding flourishing in adolescents. The objective of this study is to review the available literature on flourishing in adolescence in relation to its tie with other constructs, its study in different contexts and the way it has been operationalized. The selection of the studies was conducted in two phases. First, it was verified that the exclusion and selection criteria were met. Then, an evaluation of the quality of the pre-selected studies was carried out. The data were synthesized through the thematic synthesis method. For the results, 28 empirical studies were selected. Four thematic axes were identified: (a) Flourishing in different contexts, (b) Flourishing in regards to other results and positive psychological characteristics and/or their negative counterpart, (c) Flourishing and psychosocial vulnerability, and (d) indicators for the evaluation of flourishing. Suggestions are provided with the goal of consolidating the science of human flourishing.

For decades, psychology has focused on the detection and the approach to different psychological pathologies. However, in the last decades, new models have emerged, with the interest of understanding the optimal psychological functioning. This focus shift has meant an enrichment of the traditional model centered on the deficit (Oliva Delgado, 2015). In turn, the World Health Organization (2016) indicated that mental health does not simply refer to mild or non-existent symptomatology, but that it involves a state of well-being in which a person reaches his or her potential, is capable of facing the different situations in life and successfully contributes to the development of his or her community.

In this context, the interest in studying well-being was renewed, which in the field of psychology had its origin in two philosophical traditions. On one hand, the hedonic tradition, according to which pleasure, happiness and enjoyment are indicators of well-being. On the other hand, the eudaimonic tradition, focused on the development of one’s own potential and self-fulfillment. Currently, it is considered that although both approaches would represent aspects of well-being that are empirically different, the two would be related conceptually and would be complementary (Disabato et al., 2016).

Emergent research has given place to new theories which offer a complex perspective of well-being known as flourishing. Although there is no consensus regarding its definition, it is stated that it would be characterized for being a dynamic phenomenon with intrinsic value which would involve the continuous updating of the human potential, giving place to an optimal state of mental health (Wolbert et al., 2015). Currently, there are four major theoretical approaches that may be identified, which explain human flourishing (Agenor et al., 2017).

One of the models was presented by Diener et al. (2010), who suggest that flourishing encompasses the socio-psychological prosperity of individuals and consists of life purpose, positive relationships, engagement, competence, self-esteem, optimism and the contribution to the well-being of others. This proposal has been operationalized through the Flourishing Scale (Diener et al., 2010).

According to Huppert and So (2013), flourishing may be understood as the opposite extreme of the anxiety and depression disorders. It would consist of ten characteristics, these being positive emotions, *engagement* and purpose as core aspects; and self-esteem, optimism, resilience, vitality, self-determination, emotional stability and positive relationships as complementary aspects.

On his part, Keyes (2002, 2007) developed a model according to which there would exist a continuum representing the presence or absence of mental health. The latter would extend from states of listlessness, characterized by the absence of emotional, psychological and social well-being, to the flourishing of positive mental health, a state in which the three dimensions mentioned before are fully expressed. This proposal has been operationalized through the *Mental Health Continuum* (Keyes, 2002) and its brief version (Keyes et al., 2008).

Lastly, Seligman (2011) proposed the PERMA model, suggesting that flourishing takes place when a person experiences positive emotion, has a clear meaning and purpose in life, enjoys healthy relationships, gets involved and enjoys activities he or she is interested in, and achieves his or her personal goals. His model has been operationalized through the PERMA Profile Scale (Butler & Kern, 2016).

From an analysis of the evolution of the construct, Agenor et al. (2017) affirm that there are six basic attributes which overlap in the four theoretical approaches mentioned: meaning, positive relationships, engagement, competence, positive emotion and self-esteem. The authors mention that positive emotions and self-esteem were included due to the support of the majority. Lastly, they highlight that while five of the attributes are characteristic of eudaimonics, the positive emotion is the only one of hedonic nature.

According to findings, flourishing is a promising construct when assessing human well-being, mainly due to its relation with various indicators of positive adjustment and development. More specifically, stemming from the operationalization of Diener et al.’s (2010) model, it was found that flourishing is linked to academic performance (Datu, 2018), to creativity (Conner et al., 2018), to self-compassion (Satici et al., 2013) and it reduces the risk of suicidal thoughts (Rey et al., 2019). On the basis of the model proposed by Keyes (2002), it was suggested that positive mental health, understood as flourishing, is associated with social support (Schotanus-Dijkstra et al., 2016), with prosocial behavior (Nelson et al., 2016), with the decrease of several risk behaviors and with the implementation of a healthy lifestyle (Sofija et al., 2020). In addition, it was found that flourishing reduces the risk of mood disorders such as anxiety and depression (Doré et al., 2020; Schotanus-Dijkstra et al., 2017). Lastly, on the basis of the conceptualization and operationalization of the PERMA model (Butler & Kern, 2016), it has been indicated that flourishing is associated with gratitude, optimism, self-esteem and happiness (De Carvalho et al., 2021), with character strengths (Wagner et al., 2020) and with physical health and satisfaction with life, among other indicators of mental health (Butler & Kern, 2016).

Despite the increase observed in the number of studies about adult flourishing, empirical findings regarding flourishing in adolescents are still scarce (Witten et al., 2019).

Adolescence is a period of transition that usually has its onset in puberty, around the age of 10, and concludes around the age of 20, with the culmination of physical growth and the resolution of certain tasks, such as the consolidation of the body image, the development of a value system and the implementation of a life project (Gallegos, 2013; Güemes-Hidalgo et al., 2017; World Health Organization, 2014). Because of the particular characteristics of this stage, it is necessary to promote research that enables the understanding of well-being from a developmental perspective. In this sense, several studies point to a pattern of decrease in flourishing during mid-adolescence and late adolescence, which could be attributed to expected developmental processes (Romano et al., 2020).

It is worth mentioning that, for a long time, adolescence has been conceptualized as a moment of vulnerability that results from physical, psychological and social changes that are taking place and that are often perceived as stressful (Cossio-Bolaños et al., 2015), leading to the development of conflictive and maladaptive behaviors (Marshall, 2014; Nixon, 2014; Nixon & McClain, 2010; Tang et al., 2014; Ybarra & Thompson, 2018). However, it must be acknowledged that this stage represents a decisive period for the exploration of potential and the strengthening of those factors that foster prosperity (Kern et al., 2016; O’Connor et al., 2017).

On the other hand, the action of considering the focus of well-being in adolescence has the potential to complement and strengthen the traditional interventions focused on the reduction of psychopathological symptomatology. In the same way, studies indicate that positive mental health during adolescence provides a solid foundation that enables adolescents to face the challenges that come as they enter adulthood in an adaptive way (Hoyt et al., 2012; O’Connor et al., 2017). Finally, it is fundamentally important to deepen in the knowledge of this construct due to its impact in the design of policies which guarantee the fulfilment of human rights and promote the integral mental health of youth (Reinhardt et al., 2020). In view of the above, the objective of this work is to identify and review the available literature on flourishing in adolescence, focusing on its tie with other constructs, its study in different contexts, and the available operationalization proposals.

## Methodology

### Design

This study constitutes a classic theoretical study (Montero & León, 2007). For its elaboration, the guidelines of the PRISMA (Preferred Reporting Items for Systematic Reviews and Meta-Analyses) declaration (Urrútia & Bonfill, 2010) were taken into consideration. The steps followed in this revision were: (a) delimitation of the topic, (b) definition of the search strategy and the exclusion and selection criteria, (c) data search and storage, (d) selection of articles gathered after following the criteria, (e) evaluation of the quality of the pre-selected articles, (f) data mining, and (g) data synthesis and interpretation (Perestelo-Pérez, 2013).

### Search Strategy

The search of indexed articles in scientific journals was conducted using the key words “flourish” or “flourishing”; “adolescence” or “adolescents” and “mental well-being” or “mental health” and their respective combinations, both in Spanish and in English, in the following data sources: SciELO, Redalyc, EBSCO, PubMed, ScienceDirect, Google Scholar/Google Académico and Research Gate. The exclusion and selection criteria were taken into consideration when identifying the texts. In addition, a backward search was carried out reviewing the reference lists of the documents selected in the previous step with the purpose of identifying references of interest.

### Exclusion and Selection Criteria

Those works which met the following criteria were included: (a) works published between 2010 and 2020, (b) in Spanish or English, (c) which make explicit reference to one of the flourishing models or which contain the words “flourish” or “flourishing” or their equivalents in Spanish in the title, in the abstract or in the key words, (d) research articles of empirical nature, and (e) works that are studies about adolescent flourishing (participants aged between 10 and 20).

The following studies were excluded: (a) those published before 2010 or after 2020, (b) in languages other than Spanish or English, (c) which approach the topic in an unclear or tangential way, (d) conducted in other age groups, (e) non empirical articles, that is to say, theoretical articles, dissertations, books, book chapters, etc.

### Selection of Studies and Quality Evaluation

With the aim of limiting the bias of inclusion, the selection was carried out with the agreement of two independent reviewers and it took place in two moments. In the first instance, the selection of all those publications which met the selection criteria was considered. Then, in a second instance, it was examined whether the works met the necessary quality standards.

The quality of the studies which met the selection criteria was evaluated through a scoring system based on an overall assessment of nine domains (Long & Godfrey, 2004). The two evaluators pointed the quality of each study independently and then compared the evaluations. In case of discrepancies, the final selection of the article under consideration was discussed and decided. Each study received a score of 1 to 9 and was later classified into one of the following categories: low quality (score of 1 to 3), average (score of 4 to 6), and high (score of 7 to 9).

### Data Mining

For data mining, each of the publications was objectively described, based on eleven categories (Pires et al., 2015): language of the publication, country of the university of the first author, year of publishing, design, method of analysis, type of study, data collection material, instruments, number of participants, age and profile of the participants.

### Data Analysis and Synthesis

The data analysis and synthesis were conducted using the thematic approach proposed by Lucas et al. (2007), consisting of the grouping of selected studies into different topics. The thematic synthesis offers the possibility of drawing conclusions from the analysis of heterogeneous studies, taking the common elements as axis.

In this step, the collected data were assessed using the information gathered through the eleven categories used for data mining, and taking into consideration the general objective of the study. Each researcher conducted the initial analysis independently, reviewing and doing the thematic classification according to the identification of the main emerging topics. Subsequently, the topics identified by each researcher were compared and a consolidated list was created. Then, a grouping of topics around common dimensions was made. This last process was repeated until both researchers agreed that all the studies could be interpreted within the thematic dimensions proposed.

## Results

### Search Results

**Figure 1:**
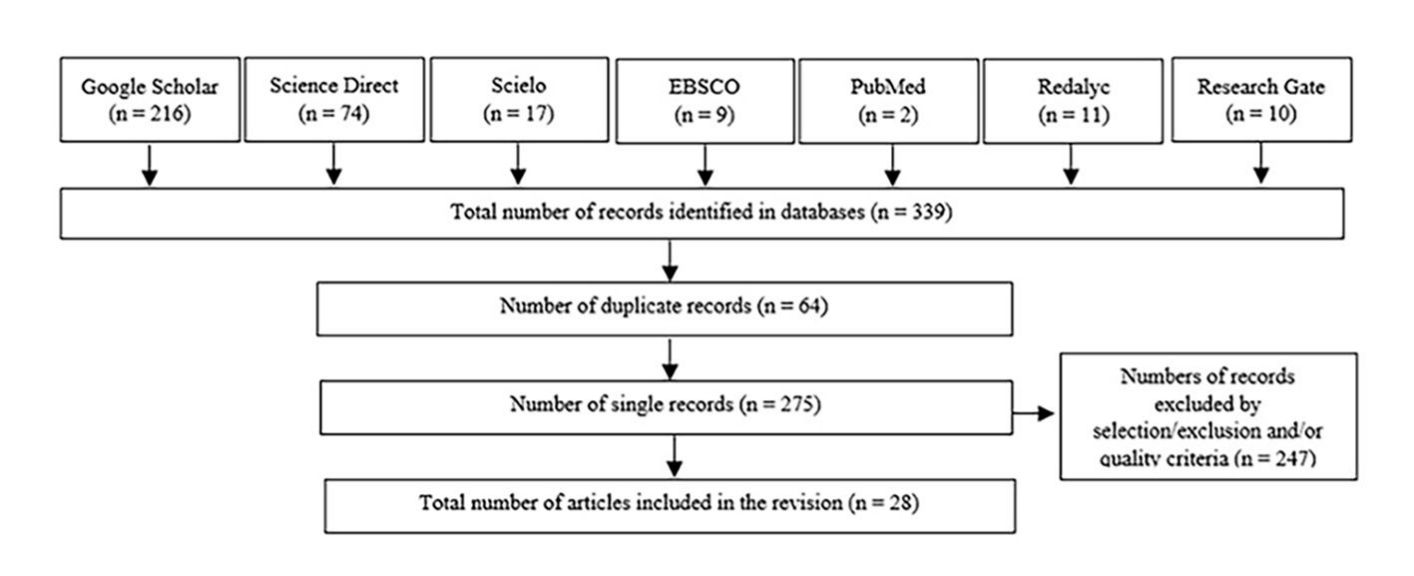
Search Stages

The initial search gave a total of 339 publications. After deleting the duplicate titles, applying the exclusion and selection criteria and analyzing the quality of the studies, 28 publications were selected to conform the present revision (see Figure 1).

### Quality Assessment

All the articles included in the previous point were submitted to quality assessment. As was mentioned before, in the Methodology section, nine domains derived from the Long and Godfrey (2004) scale were assessed. For each of the criteria that was satisfactorily met, a point was added. In this way, the articles were classified into the categories of low (scores between 1 and 3), medium (scores between 4 and 6) and high quality (scores between 7 and 9), based on the total points. While 25 of the assessed articles showed a score between 7 and 9, corresponding to an overall assessment of high quality, the other three showed average quality, with a score between 4 and 6 (see Table 1).

**Table 1:** Matrix of Articles Included in the Review

Authors	Year of publication	Geographical region	Objective	Design	Type of study	Instruments of data collection	*n,* Age	Main results	Quality assessment
Bethell, Gombojav, & Whitaker	2019	United States	To determine the prevalence and the predictors of flourishing in children and adolescents. ^c^	Cross-sectional	Explanatory	Web survey	51156 parents	The prevalence of flourishing was 40.3%. Flourishing was associated with higher levels of resilience and family connection.	High
Burke & Minton	2019	Ireland	To know the effect of flourishing, the differences according to age and gender, and the contribution of character strengths to adolescent flourishing. ^b^	Cross-sectional	Explanatory	Self-reports	2822 adolescents, Ages 12 to 19	Flourishing decreased with age and women reported lower levels than men. The under-utilization of character strengths predicted lower levels of prosperity.	Average
Butler, Patte, Ferro, & Leatherdale	2018	Canada	To examine whether depression and anxiety are associated with the use of cannabis and to know if flourishing moderates these associations. ^b^	Cross-sectional	Explanatory	Self-reports	8179 adolescents, Secondary school students	Associations between depression, anxiety and use of cannabis were not significant when considering the moderating role of flourishing. However, negative relations were detected between flourishing and cannabis use.	High
Datu	2018	Philippines	To examine the relation between flourishing and perceived academic achievement, academic performance, behavioral engagement and emotional engagement. ^b^	Cross-sectional	Explanatory	Self-reports	424 adolescents (*M* = 18.49, *SD* = 1.45); 525 adolescents (*M* = 13.85, *SD* = 1.23)	Flourishing predicted academic performance positively, after controlling age, gender and subjective well-being. Flourishing positively predicted academic achievement, behavioral engagement and emotional engagement of adolescents.	High
Duan & Xie	2019	China	To assess the psychometric properties of the Flourishing Scale (Diener et al., 2010) in a sample of adolescents from China. ^d^	Cross-sectional	Instrument building	Self-reports	766 adolescents, Ages 12 to 17 (*M* = 15.18, *SD* = 1.66)	The scale presented a one-dimensional structure and adequate psychometric properties.	Average
Guo, Tomson, Guo, Li, Keller, & Söderqvist	2015	China	To assess the psychometric properties of the MHC-SF (Keyes et al., 2008) in adolescents from China, providing evidence of validity and invariance by gender and age. ^d^	Cross-sectional	Instrument building	Self-reports	5399 adolescents, *(M* = 15.13, *SD* = 1.56)	The MHC-SF presented a three-factor structure, adequate psychometric properties, and metric and setting invariance through gender and age.	High
Kern, Benson, Steinberg, & Steinberg	2016	Australia	To develop the EPOCH Measure (Kern et al., 2015) and to assess its psychometric properties. ^d^	Cross-sectional	Instrument building	Self-reports	4480 adolescents, Ages 10 to 18	The EPOCH Measure presented a five-factor structure and adequate psychometric properties.	High
Kern, Waters, Adler, & White	2015	Australia	To test the PERMA multidimensional theory empirically, proposing a well-being questionnaire that operationalizes this model. ^d^	Cross-sectional	Instrument building	Self-reports	516 adolescents, Ages 13 to 18	Factorial analyses identified four of the five factors from the PERMA model and two factors of discomfort.	High
Kim, Jang, & Kim	2020	United States	To examine the relation between flourishing and different socio-ecological variables in children and adolescents. ^a^	Cross-sectional	Explanatory	Phone survey	45309 adolescents, Ages 10 to 17 (*M* = 13.63, *SD* = 2.35)	Several multilevel socio-ecological factors (individual characteristics, parents’ capability and family functions) were significantly linked to adolescent flourishing.	High
Kwong & Hayes	2017	United States	To assess the impact that adverse experiences in childhood have on the health and flourishing of children and adolescents. ^c^	Cross-sectional	Descriptive	Phone survey	The size of the sample is not reported, Ages 6 to 17	Significant differences in flourishing were observed according to the number of adverse family experiences. Social connection would foster flourishing among children with more than three adverse experiences.	High
Lim	2014	South Korea	To assess the psychometric properties of the MHC-SF (Keyes et al., 2008) in adolescents from South Korea. ^d^	Cross-sectional	Instrument building	Self-reports	547 adolescents, Ages 14 to 17 (*M* = 16.08, *SD* = 0.34)	The MHC-SF presented a two-factor structure and adequate psychometric properties.	High
Nabors, Merianos, Vidourek, King, Rosen, Zhang, & Swoboda	2016	United States	To examine flourishing among adolescents with and without asthma, and the impact of bullying and mood difficulties. ^a^	Cross-sectional	Explanatory	Web survey	Parents of 28721 adolescents aged 13 to 17	Adolescents with asthma presented lower flourishing than those without asthma. Adolescents with asthma who experienced bullying and feelings of sadness had lower flourishing.	High
O’Connor, Sanson, Toumbourou, Norrish, & Olsson	2017	Australia	To examine the long-term implications of adolescent flourishing in the transition to adulthood. ^b^	Longitudinal	Explanatory	Self-reports	999 adolescents, Ages 15 to 16 (first evaluation) and 27 to 28 (second evaluation)	Adolescent flourishing was associated with indicators of professional progress and the taking of civic responsibilities a decade later.	High
Orkibi, Hamama, Gavriel-Fried, & Ronen	2018	Israel	To assess a model that describes the underlying mechanism of adolescent development from a positive psychological perspective. ^b^	Cross-sectional	Explanatory	Self-reports	807 adolescents, Ages 12 to 15	A direct positive link was observed between the abilities of self-control and the positivity rate, as well as an indirect link through the social support adolescents perceived from their parents and peers.	High
Ortiz, Gutiérrez & Proestakis	2020	Chile	To adapt and assess the psychometric properties of the EPOCH Measure of Adolescent Well-Being (Kern et al., 2015) in adolescents from Chile. ^d^	Cross-sectional	Instrument building	Self-reports	1558 adolescents, Ages 11 to 18	The EPOCH presented a five-factor structure and acceptable psychometric properties.	Average
Reinhardt, Horváth, Morgan, & Kökönyei	2020	Hungary	To assess the psychometric properties of the MHC-SF (Keyes et al., 2008) and to identify well-being profiles in adolescents from Hungary. ^d^	Cross-sectional	Instrument building	Self-reports	1572 adolescents, Ages 11 to 20 (*M* = 15.39, *SD* = 2.26)	The MHC-SF presented a bifactorial structure. Invariance was observed through gender. Four flourishing profiles were identified: flourishing, moderate mental health, languishing, and emotionally vulnerable.	High
Rey, Mérida-López, Sánchez-Álvarez, & Extremera	2019	Spain	To assess the mediating role of flourishing in the relation between emotional intelligence and the risk of suicide among adolescents who are victims of bullying. ^c^	Cross-sectional	Explanatory	Self-reports	494 adolescents, Ages 12 to 17 (*M* = 14.55, *SD* = 1.67)	Through flourishing, emotional intelligence was linked to a decrease in the risk of suicide.	High
Romano, Ferro, Patte, Diener, & Leatherdale	2020	Canada	To examine the psychometric properties of the Flourishing Scale (Diener et al., 2010) and the invariance in two samples of adolescents from Canada. ^d^	Cross-sectional	Instrument building	Self-reports	74501 adolescents, Ages 12 to 19	The scale presented good psychometric properties, a strong validity convergent with other measures of well-being, and invariance through gender and ethnic-racial identity.	High
de Carvalho, Salgado Pereira, Marques Pinto, & Maroco	2016	Portugal	To analyze the psychometric properties of the MHC-SF (Keyes et al., 2008) in children and adolescents from Portugal. ^d^	Cross-sectional	Instrument building	Self-reports	216 adolescents, Ages 10 to 14 (*M* = 11, *SD* = 1.21)	The scale presented a three-factor structure and satisfactory psychometric properties. In addition, invariance was observed through gender and age.	High
Setyandari	2019	Indonesia	To adapt the EPOCH Measure (Kern et al., 2015) for adolescents from Indonesia. ^d^	Cross-sectional	Instrument building	Self-reports	514 adolescents, Ages 12 and 13	The translated scale presented acceptable psychometric functioning.	High
Singh & Raina	2020	India	To assess the psychometric properties of the Hindi and English versions of the PERMA Scale (Kern et al., 2015) and the WEMWBS Scale (Tennant et al., 2007) in adolescents from India. ^d^	Cross-sectional	Instrument building	Self-reports	1288 adolescents, Ages 13 to 18 (*M* = 15.27, *SD* = 1.08)	The scales presented adequate psychometric properties. Men experienced more positive emotions and engagement than women and students from private schools, and adolescents who were from rural areas and younger presented higher scores in positive factors of mental health.	High
Singh, Bassi, Junnarkar, & Negri	2015	India	To estimate the prevalence of flourishing and to examine its associations with mental anguish and psychosocial functioning, taking the age and the gender into account. ^b^	Cross-sectional	Comparative	Self-reports	539 adolescents, Ages 13 to 18 (*M* = 15, *SD* = 1.4)	The percentage of flourishing participants was 46.4%. Women and younger adolescents presented more flourishing than men and older adolescents. Flourishing youth reported lower prevalence of depression and of adjustment difficulties, and higher prosocial behavior.	High
Singh, Junnarkar, & Jaswal	2016	India	To assess the psychometric properties of the Flourishing Scale (Diener et al., 2010) and the SPANE (Diener et al., 2010). ^d^	Cross-sectional	Instrument building	Self-reports	789 participants, Ages 18 to 65 (*M* = 25.64, *SD* = 6.76); 2608 adolescents, Ages 11 to 17 (*M* = 14.95, *SD* = 1.45); 786 participants, Ages 18 to 56 (*M* = 24.56, *SD* = 3.87)	Scales presented satisfactory psychometric properties. The Flourishing Scale presented a one-factor structure and the SPANE presented a two-factor structure.	High
Skrzypiec, Askell-Williams, Slee, & Rudzinski	2016	Australia	To analyze the flourishing of students with special educational needs. ^a^	Cross-sectional	Comparative	Self-reports	1930 adolescents, Ages 13 to 15 (*M* = 13.7, *SD* = 1.4)	Students with special educational needs reported lower levels of flourishing than their peers who did not have special needs.	High
Odar Stough, Nabors, Merianos, & Zhang	2015	United States	To examine the flourishing of adolescents with convulsive disorders. ^a^	Cross-sectional	Explanatory	Phone survey	23799 parents of adolescents aged 14 to 17	Adolescents with convulsive disorders presented lower flourishing than their peers, and their level of flourishing was predicted by the seriousness of their symptoms, their age, their ethnicity, their gender, and their parents’ rage.	High
Sulistiowati, Keliat, Wardani, Aldam, Triana, & Florensa	2019	Indonesia	To describe the psychological, emotional and social well-being of adolescents from Indonesia. ^a^	Cross-sectional	Descriptive	Self-reports	972 secondary school adolescent students	Adolescents presented high levels of psychological, emotional and social well-being, and it was observed that 46.3% had flourishing mental health.	High
van Schalkwyk & Wissing	2010	South Africa	To examine the flourishing of adolescents from South Africa. ^a^	Mixed methods	Descriptive	Self-reports and structured interviews	1116 adolescents, Ages 15 to 17	Adolescents suggested that flourishing is characterized by leading a meaningful life, having positive social relationships, being a role model, presenting high levels of self-esteem, adaptive coping, positive emotions and gratitude.	High
Venning, Wilson, Kettler, & Eliott	2013	Australia	To describe the prevalence of flourishing in adolescents from Australia. ^b^	Cross-sectional	Descriptive	Self-reports	3113 adolescents, Ages 13 to 17	The percentage of adolescents who presented flourishing mental health was 42%. Those who were languishing showed more behaviors which represented a risk to their health.	High

^a^ Flourishing in different contexts. ^b^ Flourishing in relation to other results and positive psychological characteristics and/or their negative counterpart. ^c^ Flourishing and psychosocial vulnerability. ^d^ Indicators for the assessment of flourishing.

### Profile of the Selected Studies

From the extracted data it was detected that, throughout the years, there was an increase in the productions, with spikes of six publications a year during 2015 and 2019, five in 2016, four in 2020 and three in 2017. In the years 2010, 2013, 2014 and 2018 there was one release per year.

Regarding the language of publication, a predominance of publications in English was observed (*n* = 27), finding only one study in Spanish. Regarding the geographic region where the university of the first author was located, Australia and the United States stood out with five publications, followed by India (*n* = 3), Canada (*n* = 2), China (*n* = 2) and Indonesia (*n* = 2). Finally, publications from Chile (*n* = 1), South Korea (*n* = 1), Spain (*n* = 1), the Philippines (*n* = 1), Hungary (*n* = 1), Ireland (*n* = 1), Israel (*n* = 1), Portugal (*n* = 1) and South Africa (*n* = 1) were identified.

Furthermore, a clear predominance of studies with cross-sectional design (*n* = 26) was found. Also, a study with mixed methods and another with longitudinal design were detected. The data analysis was predominantly quantitative (*n* = 27). Regarding the type of study, there was a higher number of psychometric works (*n* = 11), followed by explanatory studies (*n* = 10), descriptive-correlational (*n* = 5) and comparative (*n* = 2).

Studies which used self-reports as the method of data collection were frequent (*n* = 22), although other works drew on phone surveys (*n* = 3) and web surveys (*n* = 2). A study combining the administration of self-reports and the conducting of structured interviews was detected (*n* = 1). Regarding the instruments, there was a variety of them. In the case of the studies which include interviews to parents, one can identify the use of items which do not conform a scale but that work as particular indicators of one of the dimensions of flourishing.

Finally, the number of participants included in the studies conducted in adolescent population varied between 216 and 74501. In all cases, the adolescents who participated in the studies were schooled. In the case of the studies in which the parents of the adolescents were interviewed (*n* = 4), the number of participants was in the range of 4345 to 51156. Lastly, an article in which the size of the sample was not informed was detected.

### Thematic Analysis of the Selected Studies

The publications included in this revision were classified in four thematic axes.

#### Flourishing in Different Contexts

In this axis, which understands flourishing as a construct influenced by the different cultural, social and context-related aspects of the place where the adolescent develops, six articles were included.

According to Kim et al. (2020), the flourishing of American children and adolescents is associated to various socio-ecological factors. More specifically, the authors suggest that overweight, sedentary lifestyle and frequent school absences are related to lower levels of prosperity in children and youth. Regarding the impact of the family context, results indicate that those parents with good psychological and physical health, who show ability to face stress, promote the flourishing of children. Furthermore, alcohol consumption and drug use in the family context is inversely related to child-adolescent well-being. Finally, those children and adolescents who participate in different family activities and who have a good bond with their parents show higher levels of flourishing.

In another study, Sulistiowati et al. (2019) assessed the mental health of Indonesian adolescents. The authors reported that, at the moment of the evaluation, 46.3% of the participants presented flourishing mental health and male adolescents showed significantly higher scores than the females. On the other hand, van Schalkwyk and Wissing (2010) evaluated the prosperity of South African adolescents. Through structured interviews, they evaluated the notion participants had of flourishing. According to their findings, it was understood as the living of a constructive life, with purpose, enjoying positive social relationships, showing self-confidence and healthy coping.

On their part, Skrzypiec et al. (2016) analyzed the prosperity of Australian adolescents with special needs. According to their findings, the students with special educational needs perceived themselves as less flourishing than those students of normative population.

Lastly, in this thematic axis, those studies which take into consideration the impact of different physical conditions on adolescent flourishing were also included. Nabors et al. (2016) explored the prosperity of young people with asthma. According to their findings, those adolescents who suffer from asthma present lower flourishing than those who do not suffer from this condition. They also detected that, while positive coping in parents fosters the flourishing of their asthmatic child, anger and rage have a negative impact. In this line, Odar Stough et al. (2015) found that those adolescents who suffer from epilepsy show lower flourishing than their peers with no convulsive disorders.

#### Flourishing in Relation to Other Positive Results and Psychological Characteristics and/or Their Negative Counterpart

Although most literature about flourishing has focused on its association with different positive results and psychological characteristics, studies evaluating its negative association with constructs such as depression and anxiety have also been detected. In this thematic section, six articles were included.

Regarding the relationship between flourishing and constructs of pathological nature, Butler et al. (2019) examined if there was an association between depression and/or anxiety and the use of cannabis in adolescents and, also, if flourishing moderated these associations. Their results indicate that flourishing alleviates the negative association between depression and/or anxiety and the use of cannabis among young people. In addition, higher levels of flourishing would result in less use of cannabis.

In this same line, according to Singh et al. (2015), flourishing adolescents would present less prevalence of depressive symptomatology and lower psychological maladjustment, manifesting a more prosocial behavior than that of their languishing peers. On the other hand, Venning et al. (2013) found that the majority of adolescents included in their study were not striving and that these adolescents, who languished or weakened, showed higher levels of behaviors that were risky for their health.

Regarding the relationship between flourishing and other positive traits, Orkibi et al. (2018) examined a model which postulates the link between self-control, positivity and the perceived social support. According to their findings, there is a positive and direct association between the skills of self-control and the level of positivity, as well as an indirect link, through the perceived social support. The authors state that both the abilities of self-control as well as the high index of positivity, understood as the ability to experience a higher number of positive emotions than negative ones, would be indicators of flourishing in adolescents.

On the other hand, Burke and Minton (2019) evaluated the flourishing and the predicting role of character strengths in adolescents from Ireland. Results indicate that all the aspects of flourishing diminish with age. Moreover, women reported lower levels of flourishing than men. Finally, they observed that the underutilization of character strengths would predict a lower level of flourishing.

O’Connor et al. (2017) drew from the assumption that mental health during adolescence would have implications in the functioning in later periods of life. Based on their findings, high levels of adolescent flourishing would predict the establishment of a professional career, civic engagement and participation in activities of volunteering a decade later.

Finally, taking into account the importance that school life has during adolescence, Datu (2018) assessed the association of flourishing with successful academic performance. Results indicate that flourishing would predict academic accomplishments, behavioral engagement and emotional engagement of students in high school.

#### Flourishing and Psychosocial Vulnerability.

According to Bethell et al. (2019), despite the fact that knowledge about *flourishing* has increased in the last years, little is known about the flourishing of those children and youth who face unfavorable circumstances. Three published works were identified in relation to the thematic of this section.

In a study conducted in American children and adolescents, the prevalence of childhood and juvenile flourishing in terms of different levels of exposure to adverse experiences, the presence of needs of special medical care and other sociodemographic characteristics was evaluated. Results showed that the prevalence of flourishing was positively associated with higher levels of resilience and family connection (Bethell et al., 2019).

In the same line, Kwong and Hayes (2017) studied the relationship between adverse family experiences and flourishing in children and adolescents. Their findings show significant differences in flourishing according to the number of adverse family experiences endured.

Lastly, Rey et al. (2019) assessed emotional intelligence and flourishing as protective factors against the risk of suicide in victims of *bullying*. According to their findings, the level of flourishing can significantly predict the risk of suicide among the victims of school harassment, especially in those cases in which the victim shows low levels of emotional intelligence.

#### Measures for the Evaluation of Flourishing.

In this thematic axis, 12 published works were detected. These can be divided according to the three major scales which operationalize *flourishing*: the *Flourishing Scale* (FS) designed by Diener et al. (2010), the *Mental Health Continuum* (MHC) by Keyes (2002) and its short version (MHC-SF, Keyes et al., 2008), and the EPOCH scale (Kern et al., 2015).

The scale of flourishing by Diener et al. (2010) has been adapted in a sample of adolescents from China (Duan & Xie, 2019) and India (Singh et al., 2016), with a one-dimensional structure replicated in both cases. On the other hand, invariance through gender, school grade and ethnic-racial identity of the adapted version of the mentioned scale in adolescent population in Canada is reported (Romano et al, 2020).

The MHC (Keyes, 2002) has been validated in the Hungarian context for adolescent population (Reinhardt et al., 2020). According to the reported results, the adaptation of the scale would present a bifactorial structure consisting of a dominant global factor (overall well-being) and another factor of specific components (emotional, psychological and social well-being). In its short version (Keyes et al., 2008), the MHC has been adapted for adolescents from Portugal (de Carvalho et al., 2016), China (Guo et al., 2015, 2016) and South Korea (Lim, 2014).

Finally, the EPOCH scale has been designed originally by Kern et al. (2016) in order to assess the PERMA multidimensional model of well-being. Said scale has been adapted for adolescent population from Chile (Ortiz et al., 2020) and Indonesia (Setyandari, 2019). Moreover, Singh and Raina (2020) validated an additional scale for the evaluation of the PERMA model in adolescents from India, reporting good psychometric properties.

## Discussion and Final Considerations

The objective of this study was to identify and review the available literature on flourishing in adolescence, focusing on its tie with other constructs, its study in different contexts, and the available operationalization proposals.

Although one can observe an increase of publications throughout the years, research about adolescent flourishing is still scarce (Bethell et al., 2019). Moreover, a clear disproportion is identified as regards the language of publishing, with a knowledge gap about the flourishing of the Spanish-speaking adolescent population. In this sense, it is highly necessary to notice and consider the cultural differences so as to avoid the extrapolation of concepts from a hegemonic context towards populations which are poorly represented in literature (Cobo-Rendón et al., 2020; Fernandes Ferreira Lima & Araujo de Morais, 2018). On the other hand, there is numeric predominance of cross-sectional studies of quantitative nature, which reveals the need to carry out longitudinal studies that allow to know the stability of flourishing, its predictors, and to verify its implications for the development (Fernandes Ferreira Lima & Araujo de Morais, 2018).

Regarding the conceptualization and operationalization of flourishing, two situations have been detected. Although a number of publications were recovered, which worked from some of the available theoretical models (e.g. Butler et al., 2019; Reinhardt et al., 2020), other works have evaluated flourishing through particular indicators (e.g. Bethell et al., 2019). This situation makes it difficult to compare the results and it generates little clarity as regards the definition of flourishing. According to Witten et al. (2019), when the construct is understood in a vague manner, instruments which do not align with the theory are designed and used and, often, they evaluate the construct in an incomplete way. The positioning of the authors regarding the underlying models of their research is necessary, since it is based on the theoretical frame supporting the work that different explanations for the researched phenomena will emerge (Fernandes Ferreira Lima & Araujo de Morais, 2018).

On the other hand, it has been detected that in various studies researchers resort to parental evaluation of adolescent flourishing (e.g. Bethell et al., 2019; Kim et al., 2020). In relationship to this, although parents continue being involved in the lives of their adolescents, it is estimated that the global evaluation of well-being is more precise when it is performed through self-reported instruments. Moreover, it must be highlighted that, from the cognitive theory, what has more impact on the behavior of people is the self-perception of psychological resources (Ben-Arieh et al., 2014; Disabato et al., 2016; Lemos, 2013).

Just as it has been observed in this review, factors which associate with and have an impact on flourishing operate in multiple levels (Kandasamy et al., 2018; Kim et al., 2020). Studies report that the anger of parents (Nabors et al., 2016; Odar Stough et al., 2015) and the use of substances (Kim et al., 2020) is related to lower levels of prosperity in children. In contrast, good physical and mental health of the parents, their coping skills (Kim et al., 2020; Nabors et al., 2016), doing family activities and having good father-son bonds (Kim et al., 2020) has a positive impact.

As far as personal factors is concerned, the increase in age (Burke & Minton, 2019; de Carvalho et al., 2016; Romano et al., 2020; Singh et al., 2015), overweight, sedentary lifestyle and frequent school absences (Kim et al., 2020), special educational needs (Skrzypiec et al., 2016) and suffering from asthma or epilepsy (Nabors et al., 2016; Odar Stough et al., 2015) would decrease the flourishing in adolescents. However, the development of character strengths would predict the prosperity of the youth (Burke & Minton, 2019). Moreover, according to different studies, flourishing would be linked to a positive development both during adolescence (Butler et al., 2019; Datu, 2018) as well as during adulthood (Keyes, 2006; O’Connor et al., 2017).

It is worth mentioning that contradictory results were observed regarding the influence of gender on flourishing. While a few studies report that boys enjoy more flourishing (Burke & Minton, 2019; Romano et al., 2020; Sulistiowati et al., 2019), others suggest that girls reach higher scores (Singh et al., 2015) or that there are no significant differences (de Carvalho et al., 2016; Reinhardt et al., 2020). These results could be explained based on the socio-cultural context or, even, on the social desirability linked to the evaluation of constructs of positive nature (Burke & Minton, 2019).

On the other hand, as it has been mentioned, there is a breach in research regarding flourishing of those adolescents who face circumstances of vulnerability. It is necessary to understand the factors that foster flourishing in those scenarios (Bethell et al., 2019). In connection with the above, due to the fact that the consequences that several situations of vulnerability have in adolescents are different from those in adults, it is necessary to know their impact on the future development and well-being (Cabieses et al., 2020).

Finally, it is worth mentioning that the evaluation of flourishing requires the construction and validation of instruments which are appropriate for the age and the socio-cultural context. According to Rose et al. (2017), although now there are more available scales for its evaluation, the construct has not yet been defined in a consistent manner. Having valid and reliable scales which operationalize the construct in a robust way would allow for the evaluation of flourishing in the different stages of life and would help to know the efficacy of the interventions designed to promote mental health.

It is necessary to highlight that a science which has the promotion of the integral health of people as its main goal should pay attention to the design of strategies which stimulate flourishing from an early age (de Carvalho et al., 2016). Besides, although for decades adolescence has been explained from theories centered in the psychopathology, this stage offers unique opportunities to foster the optimum development of all people (Giménez et al., 2010).

### Limitations and Future Instructions

Although this review identified 28 articles, other publications were excluded, which, even though they worked on adolescent flourishing or relevant aspects linked to it, they did not meet the selection criteria. The cut made in the choosing of the databases may have left out certain publications such as congress presentations or dissertations on the topic. Moreover, some of the studies include not only adolescents in their samples, but also children or young adults, which hinders the interpretation of the results. Finally, the parental evaluation of the construct that was proposed in some of the works may have distorted the information regarding the particular subjective experience of adolescents.

For future studies, we suggest that longitudinal designs be used as well as intervention designs which analyze flourishing through different stages of life and their positive correlates associated. Moreover, the need for research in Spanish-speaking contexts and in developing countries is highlighted, with the objective of establishing transcultural comparisons. Although a few works based on the self-assessment of flourishing were found, as well as others stemming from peer assessment, it is worth mentioning that the conducting of studies that combine both measures is recommended. Finally, the need of theoretical and methodological refinement which facilitates the understanding of attributes, antecedents and results of adolescent flourishing and that contributes to the building up of a science which conducts research regarding the possible ways towards human flourishing is noted.
